# Plant microbiomes

**DOI:** 10.1111/1751-7915.13451

**Published:** 2019-06-11

**Authors:** Lawrence P. Wackett

**Affiliations:** ^1^ Department of Biochemistry, Molecular Biology & Biophysics BioTechnology Institute University of Minnesota St. Paul MN 55108 USA

## Characteristics of plant microbiomes

https://microbiomejournal.biomedcentral.com/articles/10.1186/s40168-018-0519-z

This review article focuses on the question, how has plant domestication by humans affected the rhizosphere microbiome.

## Plant microbiome engineering

https://www.sciencedirect.com/science/article/pii/S0944501317311850

This review article deals with the potential for engineering plant microbiomes.

## Plant, root, soil microbiomes

https://microbiomedigest.com/microbiome-papers-collection/non-human-microbiome-paper-collection/plant-root-and-soil-microbiome/

This blog, or digest, lists recent papers in plant microbiome science.

## Plant microbiomes and conservation

https://www.sciencenews.org/article/plant-microbes-crops-food-endangered-species

This news article deals with the idea that engineering plant microbiomes may be applied to the goal of saving endangered plant species.

## Plant microbiomes and native plant restoration

https://academic.oup.com/bioscience/article/68/12/996/5148107

This paper deals with the concept that native mycorrhizal communities may be beneficial to successful grassland restoration.

## Wheat microbiome bacteria controlling fungus

https://www.nature.com/articles/s41467-018-05683-7

This study looks specifically at interactions between microbiome organisms, the bacterium *Pseudomonas piscum* and the plant pathogenic fungus *Fusarium graminearum*.

## Research priorities for plant microbiomes

https://journals.plos.org/plosbiology/article?id=10.1371/journal.pbio.2001793

This article reviews current plant microbiome research and formulates plans for a best path forward in future research.

## Fertilizer diminishes microbiome disease protection

https://phys.org/news/2018-07-fertilizer-microbiome-ability-disease.html

This news article describes research showing the fertilizer addition greatly perturbs plant microbiomes, making the plants less resistant to disease.

## Evolution of microbiomes

https://www.journals.uchicago.edu/doi/full/10.1086/698709

As plants have evolved over millions of years, microbiomes must have evolved in concert. This article deals with these evolutionary aspects.

## Rhizosphere vs. phyllosphere microbiomes

https://www.biorxiv.org/content/10.1101/365023v1.full

The rhizosphere refers to the root region of plant microbiomes whereas phyllosphere refers to the above‐ground portion of plants that make another habitat for microbes.

## Genomics database for plant microbiomes

https://jgi.doe.gov/functional-genomics-database-for-plant-microbiome-studies/

This page describes efforts to catalog plant beneficial bacteria to aid in understanding of the interactions and to potentially engineer plant microbiomes.

## Earth microbiome project

http://www.earthmicrobiome.org

The Earth microbiome is broader than plant systems. But plant microbiomes is one of the type of environments covered from a metagenomic perspective.

## Networking in the plant microbiome

https://www.ncbi.nlm.nih.gov/pmc/articles/PMC4752285/

This big picture article is expansive but mostly deals with ways to study associations between strains in complex plant microbiomes.

## Bayer moving ahead with plant microbiome company

https://www.agri-pulse.com/articles/10740-bayer-moving-ahead-with-plant-microbiome-company

This site describes the formation of a joint venture between Bayer and Ginkgo Bioworks to form Joyn Bio, a company dedicated to develop biological nitrogen fixation solutions for agriculture.

## Top plant microbiome companies

https://www.ventureradar.com/keyword/Plant%20microbiome

This business website highlights different companies with technology trained on understanding and engineering plant microbiomes for agriculture. The site provides a short synopsis and a link to the commercial site.

## Agbiome

https://agbiome.com

Agbiome studies bacteria in the plant microbiome in an effort to develop natural controls against agronomic pests such as pathogenic fungi, insects and mites.

## Pivot Bio

https://www.pivotbio.com

Pivot Bio is focused on engineering bacteria for continuous derepression of nitrogen fixation genes and overcoming other functional control. The major goal is to develop bacteria that associate with corn plant roots and supply nitrogen to the plant.

